# New methodology to improve tracking of Veteran overdose deaths and characterization of a population of Veteran overdose decedents in San Diego County

**DOI:** 10.1016/j.dadr.2025.100392

**Published:** 2025-11-04

**Authors:** Benjamin I. Felleman, Neal M. Doran, Octaviana Hemmy Asamsama, Elizabeth M. Oliva, Benjamin H. Han

**Affiliations:** a VA San Diego Healthcare System, San Diego, CA, USA; b Department of Psychiatry, University of California San Diego, USA; c Veterans Affairs Program Evaluation and Resource Center, Veterans Affairs Office of Mental Health and Suicide Prevention, Menlo Park, CA, USA; d Veterans Affairs Center for Innovation to Implementation, Veterans Affairs Palo Alto Health Care System, USA; e Division of Geriatrics, Gerontology, and Palliative Care, Department of Medicine, University of California, San Diego, USA

**Keywords:** Overdose Prevention, Veterans Affairs, Opioid Use Disorder, Harm Reduction, Public Health and Policy, Mortality

## Abstract

**Background:**

National trends indicate that drug-related deaths among Veterans have been increasing from 2010 to 2019. The present study involves a recent analysis of drug mortality data for a single large Veterans Affairs (VA) Healthcare System. The aims of the study included (1) the identification of VA patients with drug-related deaths, (2) patient characteristics and service utilization patterns of VA patients with drug-related deaths, and (3) the evaluation of existing internal tracking systems for monitoring drug-related deaths.

**Methods:**

This retrospective study matched VA enrollment records to San Diego County Medical Examiner (ME) data from January 2019 to June 2023. The records of individuals who died of a drug-related overdose in San Diego County were matched to VA medical records. Chart reviews were conducted to evaluate the extent to which intentional and accidental overdose events were documented in electronic medical records, and to examine demographic and clinical characteristics and healthcare utilization in Veterans who died by overdose.

**Results:**

From January 2019 to June 2023 there were a total of 140 drug overdose deaths, 91.4 % were accidental (*n* = 128) and 8.6 % were intentional (n = 12). Prior to ME data matching, VA records captured 9.6 % of accidental drug overdoses (n = 15) and 100 % of intentional drug overdoses (n = 12). Fentanyl or fentanyl analogs were involved in 37.1 % (n = 52) of intentional and unintentional drug overdose deaths with the combination of fentanyl and methamphetamine being the next most specific common cause of death (n = 30; 21.4 %). In terms of VA healthcare utilization, in the year prior to their death, 63.6 % of Veterans accessed care. Among those 89 VA patients, they most commonly utilized the emergency department (75 %) and primary care (56.2 %). Among the 20 % of Veterans with opioid use disorder (OUD), in the year prior to their death, 39.3 % were dispensed a prescription for naloxone and 35.7 % were dispensed a medication for OUD.

**Discussion:**

Comparing VA records to county ME records revealed that VA records missed over 80 % of drug-related overdose deaths—4 out of every 5 deaths. While accurate for intentional overdoses, accidental overdoses—which comprise the vast majority of drug overdose deaths—were missing over 90 % of the time. Given that drug toxicology results were consistent with county trends, this suggests that VA records severely underestimate drug overdose deaths. Approximately two-thirds of VA patients who died of drug overdose access VA and most were seen in the emergency department and over half in primary care—identifying these as important intervention targets for overdose prevention. Given the gaps in capturing drug overdose deaths, other healthcare systems looking to prevent overdose deaths, and especially other VA systems, may want to consider adopting similar methods to better capture and understand factors that impact drug overdose deaths among their patient populations.

## Highlights

• Linking VA records and local medical examiner data yielded rich circumstantial data on fatal overdoses for one VA healthcare system.
• Veterans Affairs (VA) national tracking method missed over 80 % of drug-related overdose deaths at the site of investigation.
• Fentanyl or fentanyl analogs were involved in 37.1 % of Veteran deaths at one VA Healthcare System.
• In the year before death, 63.6 % of Veterans accessed VA care,and 75 % of them used the Emergency Department.

## 1 Introduction

Drug overdose deaths in the United States (US) showed a dramatic rise over the past two decades, with the total number of deaths surpassing 100,000 in 2021 (CDC, 2023). This pattern is also evident in the veteran population, with increased numbers of veterans dying by overdose between 2010 and 2019 (Lin et al., 2019, Begley et al., 2022). While recent data show that drug overdose deaths are declining (CDC, 2025), there were still nearly 75,000 individuals dying of drug overdose in the US in the past year as of March 2025 (CDC, 2025). Key drivers of overdose deaths include stimulants and synthetic opioids (Kariisa et al., 2019).

Analyses of the National Death Index suggest that this trend is evident in U.S. Veteran populations, as rates of deaths associated with stimulants, both alone and with other substances, increased threefold from 2012 to 2018 (Coughlin et al., 2021). The Veteran Health Administration (VHA) is the largest integrated healthcare system in the US. Veterans are at elevated risk for drug-related deaths due to complex interactions across psychological, physiological, social, and structural domains (Bennett et al., 2022). Knowledge and awareness of this risk is critical for informing healthcare policy and intervention at VHA. The gold standard tracking method is the National Death Index (NDI), but access is restricted and analyses are lagged (Hess et al. (2019). Moreover, while large-scale mortality analyses can be useful from an epidemiological perspective, they may not provide insight into local real-time trends and regional differences in drug-related overdoses.

Internally, the VHA mandates reporting of all overdoses via nationally standardized clinical note templates (e.g., Suicide Behavior and Overdose Report [SBOR]). The SBOR is intended to inform and improve care for Veterans who overdose, thus ensuring clinical follow-up and review for any non-fatal overdoses and suicide behaviors. Additionally, it facilitates system-wide quality improvement efforts by allowing the review of all non-fatal and fatal events for patterns and opportunities for programmatic improvement. This approach can be limited as it relies on healthcare providers to learn of overdoses, including Veteran deaths, either through county records, police, or friends and family, and then document it in a specific note in the Veteran’s electronic medical record. The current study aimed to explore the utility of a novel method for detecting drug-related deaths via coordination between the VA San Diego Healthcare System and the San Diego County Medical Examiner’s office. The San Diego VA serves roughly 88,000 Veterans with services available to more than 200,000 Veterans who live in a county that has continued to experience psychostimulant-related overdose deaths despite stabilization of overall drug-related overdose deaths (San Diego County Health & Human Services, 2022). Our work was geared towards informing programmatic improvement and promotion of harm reduction initiatives through evaluation of intentional and accidental overdose events. We also aimed to examine demographic and clinical characteristics and healthcare utilization in Veterans who died by overdose.

## 2 Methods

We used data from the County Medical Examiner’s (CME) Office for 10,725 individuals who died from January 2019 to June 2023. This list was distilled down to 4455 deaths in which drugs were considered to be the leading contributing cause of death. The protected health information (PHI) obtained from the CME office was compared to the registry of 384,461 Veterans registered with VA San Diego Healthcare System. The two datasets were compared based on Veterans’ names, social security numbers and dates of birth. Matching required the same name and the same social security number or date of birth, as in some cases the CME registry did not include social security number. If any of these three criteria differed the records were not matched. Receipt of healthcare was not necessary for inclusion in the dataset, although Veterans who had never had VA contact would not have been included in the VA registry. Employees were excluded for privacy purposes. Institutional Review Board (IRB) exemption for this quality improvement project was obtained from the VA San Diego IRB.

The list of decedents and the list of Veterans were compared to generate a list of Veterans who died of drug overdose. We included those individuals who died by both intentional and unintentional overdose. Information from the Suicide Behavior and Overdose Summary Report (using the same timeframe) was generated for comparison purposes—this dashboard helps Suicide Prevention Coordinators, Overdose Review Teams, and facility leadership by providing aggregated data related to fatal and non-fatal suicide and overdoses. Available to VHA clinical staff, this dashboard allows staff to drill into clinical/case factor trends (e.g., method and outcome, demographics, diagnoses), patient-level data, and trends over time. Manual chart reviews were conducted to gather data about patient characteristics and healthcare utilization patterns. This process involved reviewing both electronic health record diagnoses as well as provider clinical notes. Variables tracked included mental health and substance use disorder diagnoses; past year visits to the emergency department, past year visit(s) with a primary care provider (MD or NP), past year visit(s) with a clinician in a mental health department, and past year engagement with our substance use treatment services. Housing status was inferred based on clinical documentation and evidence of past year correspondence with VA homeless outreach staff. Electronic health records were reviewed to determine both lifetime and past year receipt of medications for SUDs filled by VA pharmacies. Review was limited to care within the VA healthcare system and did not include care that could have potentially been received at other healthcare organizations. Descriptive analyses were conducted in SPSS 28.0.

## 3 Results

### 3.1 Veteran Characteristics

The matched sample included 140 Veterans who died between January 2019 and June 2023. These included both accidental (n = 128) and intentional (n = 12) drug-related overdoses. The average age was 55 (SD = 13.5), and ages ranged from 25 to 91 years old. The majority of the sample was White, Non-Hispanic. Full sample demographics are presented in Table 1. Accidental and intentional drug-related overdoses were combined for analyses to capture overall drug-related overdoses. Nearly one-third (27.9 %) of the sample were inferred to be homeless based on chart review methods. The most common psychiatric diagnosis was depressive disorder (34.4 %), followed closely by post-traumatic stress disorder (30.0 %). Over one-third (36.4 %) met lifetime criteria for stimulant use disorder, which was significantly more than the number of Veterans meeting criteria for opioid use disorder (22.1 %). SBORs were completed for 19.3 % of the sample—including 100 % completion for intentional overdoses but only 11.7 % completion for accidental overdoses.

**Table 1:** Veteran demographics (N = 140).

**Gender**				
	Male		114 (92.7 %)	
	Female		7 (5.7 %)	
	Unknown		2 (1.6 %)	
**Age at time of death**			M(SD) = 55 (13.5); range = 25–91	
	18–29		4 (2.9 %)	
	30–44		32 (22.9 %)	
	45–64		63 (45.0 %)	
	65 +		41 (29.3 %)	
**Race/Ethnicity**				
	White, Non-Hispanic		92 (71.4 %)	
	White, Hispanic		8 (5.7 %)	
	Black/African American		22 (15.7 %)	
	Native Hawaiian or Pacific Islander		5 (3.6 %)	
	Asian		2 (1.4 %)	
	American Indian or Alaskan Native		1 (.7 %)	
	Unknown		10 (14.0 %)	
**Confirmed Homeless**			39 (27.9 %)	
**Mental Health Diagnoses (many with multiple)**				
		Depressive Disorders	48(34.3 %)	
		Posttraumatic Stress Disorder	42 (30.0 %)	
		Anxiety Disorders	17 (12.1 %)	
		Schizophrenia Spectrum Disorder	15 (10.7 %)	
		Bipolar Spectrum Disorder	12 (8.6 %)	
		Unspecified Psychosis	7 (4.0 %)	
		Attention Deficit Hyperactivity Disorder	4 (2.9 %)	
		Insomnia Disorder	5 (3.6 %)	
		Personality Disorder	5 (3.6 %)	
		No Diagnoses or Records	29 (20.7 %)	
**SUD Diagnoses**				
		Stimulant Use Disorder	51 (36.4 %)	
		Opioid Use Disorder	31 (22.1 %)	
		Alcohol Use Disorder	7 (5.0 %)	
		Cannabis Use Disorder	3 (2.1 %)	
		Cocaine Use Disorder	1 (.7 %)	
**SUD Diagnoses in Remission**				
		Alcohol Use Disorder, in Remission	15 (10.7 %)	
		Stimulant Use Disorder, in Remission	8 (5.7 %)	
		Opioid Use Disorder, in Remission	6 (4.3 %)	
		Cocaine Use Disorder, in Remission	5 (3.6 %)	

SUD =  Substance Use Disorder

Table II.

### 3.2 Healthcare utilization

Of the 140 deceased Veterans, 89 (63.5 %) had accessed care from the VA within the past year. The most commonly accessed setting was the Emergency Department (75.0 %). This was followed by Primary Care, with 56.2 % having at least one primary care visit in the 12 months prior to death. Outpatient Mental Health was the third most accessed department (47.2 %). The remaining services examined included SUD treatment through mental health (33.7 %), emergency mental health walk-in clinic (25.8 %), and inpatient mental health (14.6 %). Seventy-eight (55.7 %) had a lifetime history of involvement in SUD treatment at the VA.

### 3.3 Medications for Opioid Use Disorder (MOUD)

A total of 20.0 % of the deceased Veterans had a documented history of opioid use disorder. Of these individuals, 11 (39.3 %) had received a prescription for naloxone, which was predominantly prescribed by mental health providers. Ten Veterans had received any medications for OUD (MOUD), which included buprenorphine (n = 5), methadone (n = 4), and naltrexone (n = 1). Mental health providers also prescribed the vast majority of MOUD (90 %).

### 3.4 Drug toxicology results

Drug toxicology results found that over half of Veterans (56.4 %) had opioid toxicity at their time of death, with 37.1 % having detectable levels of fentanyl. Stimulant toxicity was identified among 52 % of Veterans, 37.9 % involved methamphetamine and 7.8 % involved cocaine. Alcohol toxicity was identified in 15.0 % of Veteran drug overdose deaths. The most common toxic combination in Medical Examiner reports was methamphetamine combined with fentanyl. This accounted for 21.4 % of deaths. Miscellaneous cardiopulmonary or other medical conditions with drug contribution were deemed the second most common cause of death (17.9 %). The third most prevalent cause of death was methamphetamine alone, resulting in 15.7 % of total deaths. Additional drug toxicology results and causes of death are presented in Table 2.

**Table 2:** Toxicology report and causes of death (n = 140).

**Drug and Alcohol Toxicity**		
	Any opioid toxicity at time of death Any fentanyl toxicity at time of death	79 (56.4 %) 52 (37.1 %)
	Any stimulant toxicity at time of death Any methamphetamine toxicity at time of death Any cocaine toxicity at time of death	64 (45.7 %) 53 (37.9 %) 11 (7.8 %%)
	Any alcohol toxicity at time of death	21 (15.0 %)
**Causes of Death**		
	Methamphetamine + Fentanyl	30 (21.4 %)
	Methamphetamine alone	22 (15.7 %)
	Fentanyl alone	19 (13.6 %)
	Prescription Opioids	13 (9.3 %)
	Prescription Drugs (non-opioids)	12 (8.6 %)
	Methamphetamine + opioid (non-fentanyl)	4 (2.9 %)
	Heroin + Methadone	1 (.08 %)
	Heroin	2 (1.4 %)
	Heroin + Methamphetamine	3 (2.1 %)
	Cocaine	5 (3.6 %)
	Cocaine + Fentanyl	6 (4.3 %)
	Prescription Opioids + Fentanyl	1 (.08 %)
	Other Mixed Drugs Complications	5 (3.6 %)
	Cardiopulmonary related deaths with unknown drug contribution	25 (17.9 %)

## 4 Discussion

The current study utilized a novel method to perform a local analysis of drug-related overdose deaths among Veterans enrolled at VA San Diego that occurred within San Diego County between January 2019 and June 2023. We matched VA medical records with the local county medical examiner’s office to examine patient mortality. Chart reviews were then conducted with the goals of 1) identifying clinical correlates and opportunities for intervention, and 2) comparing mortality findings to existing VA data from the SBOR summary report. Our decision to complete chart reviews as opposed to extracting data (e.g., ICD-10 diagnosis codes) was a strength of the study. This allowed for a careful review of medical record narratives instead of relying on provider coding, which can be prone to error.

Consistent with national trends, we found fentanyl to be an increasingly frequent cause of death year over year. The combination of methamphetamine and fentanyl was the most frequent cause of death. Veterans at highest risk for drug-related overdose deaths were primarily White, Non-Hispanic, middle-aged (45 +) males. A third of the sample were older adults (65 +), which is unsurprising given the aging Veteran population and the increasing trend nationally (Humphreys and Shover, 2023). Older adults are particularly at risk for overdoses given physiological changes related to aging and co-occurring chronic diseases, including interactions with stimulants and existing cardiovascular disease. Interventions that address SUD in the setting of medical multimorbidity are needed to reduce the harms of drug use in this population (Han et al., 2019). Additionally, nearly 30 % of the sample were veterans inferred to be experiencing homelessness, which also represents a population at high risk for overdose (Fine et al., 2022). This highlights the importance of delivering harm reduction interventions (including naloxone and fentanyl test strips) and evidence-based SUD treatment for this population, especially for the VA through Homeless Patient Aligned Care Teams (HPACT) and syringe services programs. Additionally, efforts should be made to expand the availability of harm reduction interventions outside of these settings, including through emergency departments, primary care, vending machines, and veteran outreach events such as VA Homeless Stand Downs and overdose awareness events (Tsai et al., 2023; US Dept. of Veterans Affairs, 2025)

We also examined treatment utilization among Veterans who had accessed care at the VA within the year prior. Consistent with findings from D′onofrio, McCormack, & Hawk (2018), the emergency department was a common touch point for individuals with opioid and stimulant use disorders. Notably, nearly one-third of Veterans who died by overdose had no past year contact with the VA. This important finding highlights the need for strong partnerships between VA and community-based prevention and intervention programs. Locally, the emergency department has been a setting we have targeted for outreach and harm reduction education. The integration of peer navigators within the emergency department could potentially bolster these efforts. Qualitative studies involving veterans with SUDs may provide better insights to improve engagement with this population in VA care to identify and remove barriers to interventions that reduce overdose risk. Moreover, it would be advantageous for future work to examine potential ways to most accurately capture non-fatal overdoses through the SBOR system, as this could serve as an important life-saving point of intervention.

A secondary focus was on identifying discrepancies between internal dashboards and county medical records. We discovered that the VHA internal dashboard missed the vast majority, 80.7 %, of drug overdose deaths. This was expected given that the dashboard relies on a process that would require a third party to communicate to the VA about a Veteran’s cause of death, and then for a provider to enter the required note in the Veteran’s medical records. These results highlight a major gap in visibility of drug overdose events and highlight the need to improve accurate monitoring of Veteran drug overdoses—both fatal and non-fatal events—to inform and improve care for Veterans. Given the lags in medical examiner determination of cause of death as well as stigma that families and Veterans may experience related to substance use and overdose, it is clear that innovative strategies will be needed to improve reporting and tracking of these events. For example, better coordinated strategies could support capturing Veteran status in mortality databases and involve coordination between county, state, and federal organizations. In California, such legislation has been proposed under Assembly Bill AB1462, which will require the California Department of Public Health (DPH) to access existing data within the electronic death registration system (EDRS) to compile a report on veteran drug overdose deaths in California and to report specified data. Even without legislation, there are processes available to help public health officials coordinate data sharing through a formal Overdose Fatality Review (Office of Financial Research, 2025).

There were limitations to this retrospective chart review study. The medical records were at times ambiguous. Toxicology reports could point to drug-related contributors, but the overall reports were not detailed enough to connect the substances to interactions with other chronic health conditions. A more granular level of analysis of multimorbidity and drug interaction would be important but was outside the scope of this program evaluation. Another limitation was that our review of Veteran mortality was descriptive in nature and limited to only those Veterans who had ever enrolled in VA healthcare at the San Diego VA. The extent to which the findings from our single, but large, catchment area generalize to other VA facilities is an important point of exploration for future research and/or program evaluation. Moreover, it is likely that of the 4455 deaths in the county, at least some had served in the military but had never enrolled in VA healthcare. It is therefore possible that our existing method for tracking unintentional drug-related deaths is capturing 9 % or less of actual deaths.

There are clear clinical and public health implications to take away from this retrospective analysis. Locally, our facility has been taking advantage of the VA Emergency Medicine Addiction Hotline (VEMAH), which has been available since May 2023. We have also focused on providing harm reduction interventions through our syringe services program, which provides SUD treatment, fentanyl test strips, and naloxone. Low-barrier treatment is readily available. Continued tracking of overdose trends will be critical for assessing the effectiveness of these efforts. The integration of Natural Language Processing and other technical tools could be a more efficient process moving forward compared to the chart review method conducted.

Engaging Veterans in care will be critical given that one-third had not accessed VHA care in the year prior to their death. To that end, VA San Diego has a mobile harm reduction van that seeks to meet Veterans where they are and that specifically targets areas with high rates of overdose. Moving forward it is clear that these types of efforts as well as community partnerships to ensure care and support of Veterans across our county will be critical. Our hope is that with further education, training, and destigmatization around SUDs, more Veterans will take advantage of the life-saving treatments available to them.

## Author disclosure

This research was supported by grants through the National Institute on Drug Abuse: K23DA043651, R21DA062721, and R21DA058404 (Dr Han). The content is solely the responsibility of the authors and does not necessarily represent the official views of the National Institutes of Health.

## Declaration of Competing Interest

The authors declare that they have no known competing financial interests or personal relationships that could have appeared to influence the work reported in this paper.
